# Retinoids in Stellate Cells: Development, Repair, and Regeneration

**DOI:** 10.3390/jdb7020010

**Published:** 2019-05-24

**Authors:** Rita Carmona, Silvia Barrena, Ramón Muñoz-Chápuli

**Affiliations:** 1 Department of Animal Biology, Faculty of Science, University of Málaga, 29071 Málaga, Spain; rita@uma.es (R.C.); silviabarrenagarcia@gmail.com (S.B.); 2 Andalusian Center for Nanomedicine and Biotechnology (BIONAND), 29590 Málaga, Spain

**Keywords:** hepatic stellate cells, pancreatic stellate cells, Ito cells, retinoids, retinoic acid, tissue repair, organ regeneration, pancreatic ductal adenocarcinoma

## Abstract

Stellate cells, either hepatic (HSCs) or pancreatic (PSCs), are a type of interstitial cells characterized by their ability to store retinoids in lipid vesicles. In pathological conditions both HSCs and PSCs lose their retinoid content and transform into fibroblast-like cells, contributing to the fibrogenic response. HSCs also participate in other functions including vasoregulation, drug detoxification, immunotolerance, and maintenance of the hepatocyte population. PSCs maintain pancreatic tissue architecture and regulate pancreatic exocrine function. Recently, PSCs have attracted the attention of researchers due to their interactions with pancreatic ductal adenocarcinoma cells. PSCs promote tumour growth and angiogenesis, and their fibrotic activity increases the resistance of pancreatic cancer to chemotherapy and radiation. We are reviewing the current literature concerning the role played by retinoids in the physiology and pathophysiology of the stellate cells, paying attention to their developmental aspects as well as the function of stellate cells in tissue repair and organ regeneration.

## 1. Introduction

**Figure 1:**
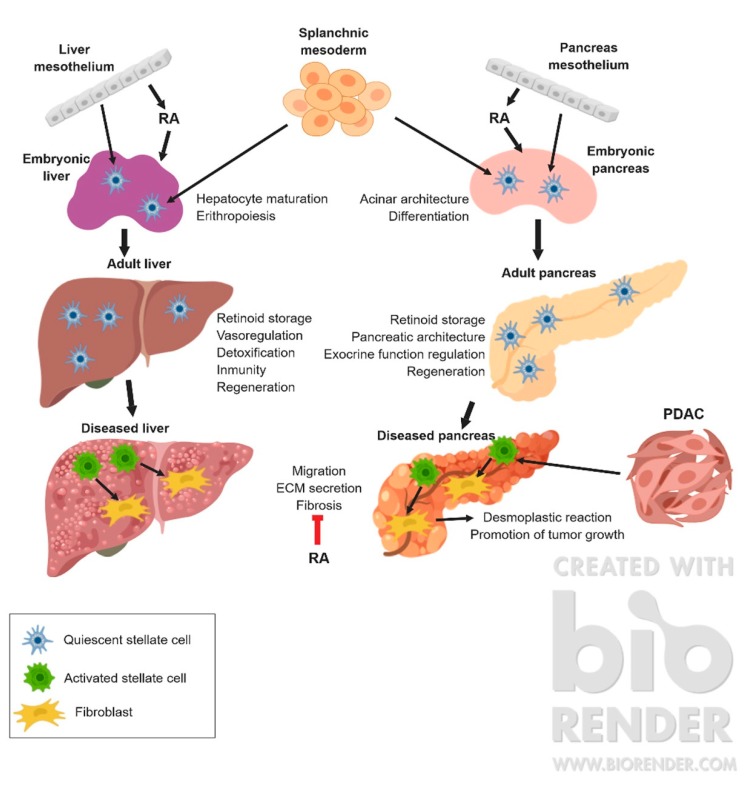
Graphical summary of the development and functions of stellate cells in physiological and pathological conditions. ECM: extracellular matrix; PDAC: pPancreatic ductal adenocarcinoma; RA: retinoic acid.

Stellate cells, also called Ito cells, were first described by Kupffer in 1876 as “*Sternzellen*”. They were overlooked for almost a century, until their rediscovery in 1971 by Kenjiro Wake [1]. We currently know that stellate cells are a type of interstitial cell mainly present in liver and pancreas, although they have also been localized in the intestine and lungs. They can be identified by the expression of desmin, endoglin, and glial fibrillary acid protein (GFAP). A main feature of stellate cells is the presence of cytoplasmic lipid vesicles where they store large amounts of retinoids. In fact, between 50% and 80% of all vitamin A in the human body is stored in hepatic stellate cells (HSCs) as retinyl esters [2]. These retinoid stores allow for easy identification of stellate cells since their exposure to ultraviolet light elicits a blue fluorescence [3]. Pancreatic stellate cells (PSCs) represent 4–7% of all the pancreatic cells, and their characteristics are similar to those of HSCs, including the accumulation of retinyl-esters in lipid vesicles and their ability to become activated [3,4]. Despite the great extent of similarity between the transcriptomes of HSCs and PSCs [5], proteome comparison reveals some differences. Proteins more abundant in HSCs are associated with protein synthesis, while PSCs show a higher abundance of proteins involved in cell structure [6]. The expression of genes related with retinoid metabolism showed no significant differences,

In pathological conditions, stellate cells from both liver and pancreas lose their lipidic droplets and transform into fibroblast-like cells, a phenotype able to synthesize large amounts of extracellular matrix, contributing to the fibrogenic response to tissue damage [7] (Figure 1).

HSCs account for 5–8% of all the hepatic cells [8]. Besides their roles in retinoid storage and fibrotic response, HSCs also participate in vasoregulation through interaction with endothelial cells, drug detoxification, immunotolerance, and maintenance of the hepatocyte population [9]. Secretion of cytokines and chemokines, activation of immune cells, capacity for antigen presentation, and autophagy activity make HSCs important regulators of liver immunology [10,11].

PSCs also display a number of relevant functions [12]. For example, PSCs maintain pancreatic tissue architecture, regulate pancreatic exocrine function, and reduce insulin expression in β-cells [13]. However, the clinically significant interaction between PSCs and cancer cells has attracted the attention of researchers in recent years, as explained below [4].

We aim to review, in this article, the relevant information about the role played by retinoids in the physiology and pathophysiology of the stellate cells from liver and pancreas, paying attention to developmental aspects as well as the function of stellate cells in tissue repair and organ regeneration.

## 2. Development of Stellate Cells in Liver and Pancreas

**Figure 2:**
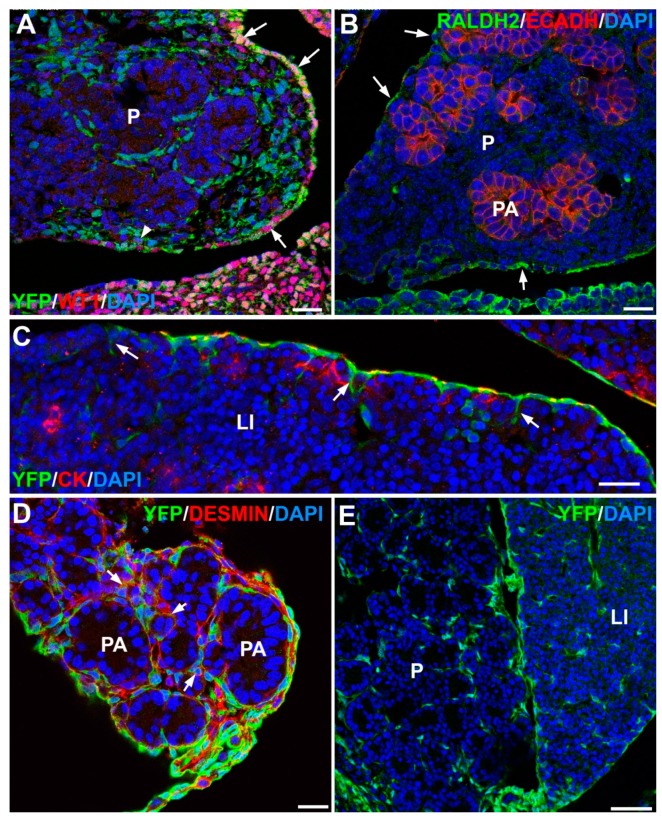
Origin of stellate cells during pancreas and liver development. (**A**) Wt1^Cre^; R26R^EYFP^ mouse embryo, stage E13.5. WT1 protein (in red) is expressed only in the coelomic epithelium of the embryonic pancreas. However, WT1-lineage cells (green) constitute most of the pancreatic stroma around the developing acini. Pancreatic stellate cells (PSCs) will develop from these stromal cells in later stages. (**B**) Mouse embryo, stage E15.5. The retinoic acid synthesizing enzyme Raldh2 (green label) is expressed in the pancreatic coelomic epithelium (arrows). Pancreatic acini (PA) are stained in red due to the expression of E-cadherin. (**C**) Wt1^Cre^; R26R^EYFP^ mouse embryo, stage E15.5. In the liver (LI), cells migrating from the coelomic epithelium (positive for cytokeratin staining in red) and expressing the WT1 lineage marker (green) also incorporate to the mesodermal compartment of the liver (arrows). (**D**) Wt1^Cre^; R26R^EYFP^ mouse embryo, stage E16.5. Colocalization of desmin (red) with the WT1 lineage marker (green) shows that part of the pancreatic stellate cells derives from the coelomic epithelium (arrows). (**E**) Wt1^Cre^; R26R^EYFP^ mouse embryo, stage E19.5. Pancreas and liver contain a similar population of WT1 lineage cells (green). Part of them have already differentiated into stellate cells. Scale bars: (**A**)–(**D**) = 25 μm; (**E**) = 50 μm.

The embryonic origin of HSCs was first related with the endoderm from a supposed common hepatoblast/HSC progenitor [14,15,16]. Another candidate was the neural crest, due to the expression of neural markers such as GFAP [17,18]. However, a mesodermal origin has been now well established (reviewed in [19]) (Figure 2). Mesenchymal cells of the septum transversum become trapped in the subendothelial space of the earliest stages of development of liver sinusoids [20]. Later, further HSC progenitors derive from cells delaminating from the coelomic epithelium of the liver, as first described by IJpenberg et al. [21] (Figure 1 and Figure 2C). These authors showed that WT1, a transcription factor expressed by the coelomic epithelium, controls the expression of the retinoic acid (RA)-synthesizing enzyme Raldh2, and this signalling axis is required for HSCs and also for proper liver development. Wt1-null mouse embryos showed reduced Raldh2 expression, less hepatoblast proliferation, and abnormal differentiation of HSCs. These findings were later confirmed by the study of Asahina et al. [22] (reviewed in [23]).

HSCs start to accumulate retinoids during foetal life, as early as E13 (stage 13 days post coitum) in rat embryos [24,25]. These authors also described a strong expression of hepatocyte growth factor and SDF1α (an important cytokine in hematopoietic stem cells homing) in HSCs, suggesting a key role in hepatic development and also in haematopoiesis, as described below. The interaction of HSCs with hepatic progenitor cells is essential for liver development and can be related with RA signalling [19]. In vitro, retinoic acid induces maturation of cultured foetal hepatocytes, increasing the production of albumin and reducing α-fetoprotein synthesis [26].

The embryonic origin of PSCs is also related, at least in part, with the coelomic epithelium of the pancreas. Between E10.5 and E15.5 in mouse embryos, this epithelium expresses WT1 (Figure 2A), allowing for tracing of the coelomic-derived cells. Transgenic mice generated by inserting a Cre recombinase expressing sequence under control of a WT1 promoter (Wt1^Cre^) can be crossed with gene-targeted mice where a stop sequence flanked by lox sites precedes a YPF cassette in the Rosa26 locus (Rosa26R^EYFP^ reporter). The offspring of these crosses, carrying both genetic modifications, will show permanent expression of YFP in Wt1-expressing cells and their lineage [27] (Figure 2A,D,E).

Using this cell tracing system, we have shown that coelomic-derived cells express Raldh2 (Figure 2B) and contribute to a major part of the pancreatic mesenchyme and the PSCs [28]. Deletion of the transcription factor WT1 between E9.5 and E12.5 results in normal dorsal pancreatic bud, but the number of acini in the ventral bud as well as the proliferation of acinar cells are significantly reduced.

Retinoid stores in HSCs can be related with developmental processes of other tissues and organs. Cellular retinol-binding protein-1 (cRBP-1) is a marker of HSCs, and the number of cRBP-1 + HSCs was used in the liver human foetuses with congenital diaphragmatic hernia to search for a correlation with the growth of liver and lungs [29]. The results showed a positive correlation between the number of cRBP-1 + HSCs and the lung weight of the side not affected by the hernia, but the lung from the affected side showed no significant correlation. The liver growth was not correlated with the number of cRBP-1 + HSC. Thus, some relationship between the hepatic retinoid stores and the lung growth may exist.

HSCs can play some role also in the foetal liver phase of definitive haematopoiesis. HSCs are topologically associated to hematopoietic sites (the space of Disse) [30]. Insulin-like growth factor 2 (IGF2) is highly expressed by murine HSCs by the time of expansion of the haematopoietic population (E12.5-E16.5) [31]. IGF2 is also secreted by hepatoblasts, and it is an essential component of the foetal liver erythropoietic niche [32,33]. Isolated adult HSCs co-cultured with Sca1+ hematopoietic stem cells support these cells in a similar way to bone marrow mesenchymal stem cells [30]. Retinoids accumulated by HSCs and retinoid X receptor α (RXRα) signalling are both required for erythropoiesis. Erythropoietin (EPO) is a direct target of RA, and it is produced by foetal hepatoblasts. EPO expression is 10 folds lower in RXRα^−/−^ foetal liver than in controls at E10, but there are no differences at E12, suggesting that RXRα mediated signalling is dispensable from E12 on [34] (reviewed in [35]).

Like HSCs, PSCs possess the mechanisms to accumulate and metabolize retinoids, bioactive molecules that are involved in both, exocrine and endocrine pancreas development [36]. Differentiation of ducts and endocrine cells during development is induced by epithelial-mesenchymal interactions where retinoic acid signalling is critically involved. Exogenous retinoic acid stimulates differentiation of duct and endocrine cells in tissue culture experiments; it promotes apoptosis of acinar cells and upregulates PDX1 expression [37]. This requirement of RA signalling for development of the pancreas does not apply to the embryonic liver [38]. Since Raldh2 is only expressed by embryonic pancreatic mesothelial cells (Figure 2B), and retinoids are stored in PSCs, these findings emphasize the importance of PSCs in pancreatic development.

β1 integrin and collagen I matrix interactions are required for maintaining foetal pancreatic stellate cell function and proliferation. In fact, culture of human foetal PSCs on collagen I stimulates their proliferation, and blocking of the β1 integrin reduces adhesion, migration and proliferation [39].

Development of stellate cells has been studied in other animal models. In zebrafish, HSCs labelled with a hand2:EGFP (enhanced green fluorescent protein) reporter colonize the liver migrating from the lateral mesoderm after the sinusoidal endothelial cells [40]. Migration is reduced if vascular endothelial growth factor is inhibited. However, HSC migration does not depend on endothelium and it occurs even in mutant zebrafish lacking of endothelial cells. In this case, HSCs abnormally associate with biliary cells.

Fibroblastoid progenitors of HSCs appear in the Disse’s space of 6-day-old chick embryos. Retinoid accumulation revealed by fluorescence of lipid droplets starts later, by 9 days of incubation. By the end of incubation, about a half of the perisinusoidal cells show retinoid-containing droplets [41].

## 3. Stellate Cells and Tissue Fibrosis

**Figure 3:**
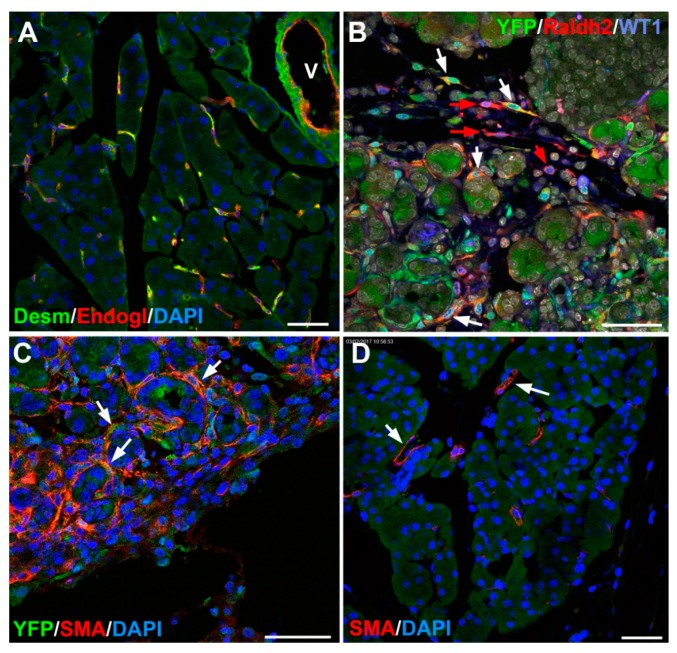
Adult stellate cells in mouse. (**A**) Pancreatic stellate cells are located around the pancreatic acini and can be identified by desmin (green) and endoglin (red) coexpression. In the vessels (V), desmin is expressed by the smooth muscle and endoglin by the endothelium. (**B**) Wt1^Cre^; R26R^EYFP^ adult mouse. After a pancreatic injury (in this case, caerulein-induced pancreatitis), stellate cells upregulate the expression of WT1 and its transcriptional target, the retinoic acid synthesizing enzyme Raldh2 (red). Some of the Raldh2 expressing cells show the WT1-lineage marker yellow fluorescent protein (YFP) (white arrows) and other are YFP-negative (red arrows), suggesting de novo expression of WT1. (**C**) Wt1^Cre^; R26R^EYFP^ adult mouse. Caerulein-induced pancreatitis. Activated pancreatic stellate cells express smooth muscle α-actin. (**D**) Normal pancreas. Smooth muscle α-actin expression is restricted to perivascular cells. Scale bars: 50 μm.

As stated in the introduction both HSCs and PSCs can be activated by extrinsic signals and transdifferentiate into a myofibroblast-like phenotype, with enhanced migratory and extracellular matrix secreting abilities and expression of smooth muscle cell α-actin [19] (Figure 3). In chronic disease, the repeated activation of stellate cells causes hepatic or pancreatic fibrosis, characterized by disruption of the normal cytoarchitecture and excessive deposition of extracellular matrix, particularly fibrillar collagens, proteoglycans, and fibronectin. Transforming growth factor-β (TGFβ) is a main activator of the stellate cell activation and the fibrogenic process, as well as the mechanical stiffness of the extracellular matrix [42]. This is clinically relevant since progressive liver fibrosis can lead to cirrhosis. Thus, HSCs have become a key cellular target for therapeutic intervention in liver fibrosis [43,44,45].

Retinoids have a strong ability to modulate the activation of stellate cells in both liver and pancreas. However, the role played by RA signalling in the process of stellate cell activation is still poorly known [19,46]. For example, it is not known why retinoid stores are lost during activation, if this loss is required for the process, or what the precise effects of retinol metabolites on stellate cells are after pancreas or liver injury [46]. According to these authors, RA released from HSCs regulates the immune response upon liver inflammation. On the other hand, it has long been known that vitamin A inhibits liver fibrosis. Reversion of liver fibrosis could be mediated by a synergistic action between RA and peroxisome proliferator-activated receptor-γ (PPAR-γ) signalling through the formation of transcriptional heterodimers between their receptors [47]. Fibrosis regression is concomitant to a reversion of HSCs to an inactive phenotype [48]. Again PPAR-γ signalling is involved in this reversion since ectopic expression of this nuclear receptor reverts cultured activated HSCs to quiescence [49]. In vitro, RA suppresses HSC proliferation [50], but divergent effects on HSCs have been attributed to the different effects of all-trans-RA (ATRA) and 9-cis-RA, as well as to differences between natural and synthetic retinoids [51,52].

In normal pancreas, retinoic acid signalling is restricted to the islets and a few exocrine cells, as shown by the retinoic acid responsive element (RARE-LacZ) murine model. After induction of pancreatitis with caerulein, the acinar cells become responsive to RA. However, when pancreatic cancer is induced, RA signalling activity is not detected in the tumours or in their precursor lesions [53]. This observation can be clinically significant, as commented below. PSCs reduce proliferation and synthesis of extracellular matrix and α-smooth actin when they are treated with ATRA or vitamin A [54,55]. Retinoic acid promotes the quiescent, non-activated state of PSCs [54,56]. Elimination of activated PSCs can also occurs by apoptosis [57]. Cellular senescence is a third, independent process in the termination of PSC activation [58]. This process had been previously involved in HSC elimination after acute liver damage [59].

All these observations about the behaviour of PSCs are important because activated PSCs promote growth of pancreatic ductal adenocarcinoma (PDAC) and the excess of extracellular matrix (called desmoplasia, desmoplastic reaction or stromal reaction to a tumour) increases the resistance of pancreatic cancer to chemotherapy and radiation. PSCs indirectly stimulate tumour growth and metastasis by promoting angiogenesis. PSCs from the primary tumour have been localized even associated to metastatic foci where they facilitate the proliferation of tumour cells (reviewed in [60,61]). Retinoic signalling and retinoid receptors are much reduced in PDAC [62]. Thus, targeting PSC activation can be considered as a therapeutic target in PDAC, and knowing better the role played by retinoids in the modulation of the quiescent/activated state must be relevant in this regard.

The Kras(G12D)/Trp53(R172H)/Pdx-1-Cre (KPC) mouse model of PDAC [63,64] has been used in several studies about the effects of retinoids on PSC/tumour cell interaction. Combined gemcitabine and ATRA treatment in KPC mice was more effective for reducing tumour growth than the use of gemcitabine alone [65,66]. Delivery of ATRA and small interfering RNA targeting HSP47 (a collagen-specific molecular chaperone) using gold nanoparticles induces PSC quiescence and reduces the desmoplastic reaction. This treatment significantly enhanced the efficacy of chemotherapeutics [67]. The effect of ATRA can be mediated by (RARβ)-dependent downregulation of actomyosin contractility, reducing the mechanical ability of PSCs for extracellular matrix remodelling [68] and release of TGFβ from its complex with latent TGFβ binding protein [69].

Open issues that deserve attention are the changes in retinoid storage and metabolism during stellate cell activation, as well as the roles of HSCs during fibrosis resolution. About the first issue, it has been shown that the stored retinols are metabolized into RA, promoting a number of reparative RA-dependent pathways in hepatocytes and also in immune cells [46].

## 4. Stellate Cells and Organ Regeneration

Besides their fibrogenic activity, activated stellate cells secrete cytokines and growth factors that promote the regeneration of both liver and pancreas. RA released by activated HSCs can have a direct mitogenic effect on hepatocytes through the RXRα receptor, contributing to tissue regeneration (Bushue and Wan, 2009) [70].

A surprising role of HSCs in hepatic regeneration was suggested by Yang et al. [71]. These authors used a fate-mapping model based on the expression of GFAP (a marker of HSCs), in mice fed with a diet that activates and expands HSCs and oval cell populations. After liver injury, HSCs downregulated GFAP, but the GFP reporter was found in highly proliferative cells that coexpressed markers of mesenchymal and oval cells. Later, GFP+ hepatocytes repopulated large areas of the liver. Similar findings were reported by using EGFP-labelled HSCs purified by retinoid-dependent fluorescence activated cell sorting and transplanted to rats that had undergone partial hepatectomy [72]. The implanted cells gave rise to mesenchymal cells, hepatocytes and cholangiocytes. Some HSCs engrafted in the bone marrow of hosts, and could be isolated and retransplanted in other rats with injured liver, where they displayed the same progenitor functions. Thus, both studies suggest that HSCs can be considered as a source of liver progenitor cells, although it is uncertain the role that RA signalling can play in the process.

PSCs also play an essential role in the regeneration of the pancreas after partial pancreatectomy. Proliferation of acinar and islet cells increases when co-cultured with activated PSCs. Interestingly, this effect is dependent of the production of extracellular matrix by the PSC. In fact, inhibition of the collagen synthesis using a specific siRNA encapsulated in a vitamin A-coupled liposome blocked this mitogenic effect [73].

As described above for HSCs, PSCs might also display a wide regenerative potential. Treatment of isolated PSCs with cytokines can induce the expression of hepatocyte markers. Transplantation of GFP-labelled, activated PSCs into partially hepatectomised rats showed differentiation into hepatocytes and cholangiocytes [74]. However, no evidence has hitherto been obtained of acinar or islet cell differentiation from PSCs.

The importance of RA signalling in pancreatic regeneration has been emphasized by our recent study on the conditional deletion of WT1 in adult mice [75]. Downregulation of WT1 in pancreas provokes a severe deterioration of the exocrine pancreas, with mesothelial disruption, E-cadherin downregulation, disorganization of acinar architecture, and accumulation of ascitic transudate. Despite this extensive damage, pancreatic stellate cells do not become activated and lose their canonical markers. We observed that pharmacological induction of pancreatitis in normal mice provokes de novo expression of WT1 in pancreatic stellate cells, concomitant with their activation (Figure 3B). When pancreatitis was induced in mice after WT1 ablation, pancreatic stellate cells expressed WT1 de novo and became activated, leading to a partial rescue of the acinar structure and the quiescent pancreatic stellate cell population after recovery from pancreatitis. We propose that WT1 modulates through the RALDH2/retinoic acid axis the restabilisation of a part of the pancreatic stellate cell population, and, indirectly, the repair of the pancreatic architecture, since quiescent pancreatic stellate cells are required for pancreas stability and repair.

## 5. Conclusion and Perspectives

In recent years both HSCs and PSCs have acquired great importance with respect to knowledge of the normal functions of the liver and pancreas, and also because of their roles in pathologies of these organs. The ability of these cells to store and metabolize retinoids seems to be highly related to their physiological and pathophysiological functions. The unique properties of these cells in the liver and pancreas homeostasis and repair raise the issue of their potential utility in cell therapy. A preclinical study has demonstrated that cotransplantation of HSCs with hepatocytes improves hepatocyte engraftment [76]. We have also described above the possibility of transdifferentiation of HSCs into hepatocytes (71,72). On the other hand, the issue of the relationships established among PSCs and pancreatic cancer cells is particularly important. Despite this significance, many uncertainties still remain, particularly about the mechanisms related with accumulation, metabolism, and release of retinoids, and the molecular regulation of activation and return to quiescence. A better knowledge of the mechanisms controlling these mechanisms could be attained using new generation technologies, such as single cell RNA sequencing, functional imaging of living systems, or development of organoids. We are aware that the research on these fields will increase the relevance of the stellate cells and the attractiveness of their potential as clinical targets.
